# Supplementary material for: Geographical Patterns of Genetic Variation in Locoto Chile (Capsicum pubescens) in the Americas Inferred by Genome-Wide Data Analysis
Source: Plants (Basel). 2022 Oct 29;11(21):2911. doi: 10.3390/plants11212911 (PMC9656212; doi:10.3390/plants11212911)
Supplement: Supplementary file 1 [file plants-11-02911-s001.zip › plants-1977816-supplementary.pdf]

Figure S1. Morphological diversity among the studied *C. pubescens* accessions. Representative images showing: (**a**) corolla colour and number of pieces, (**b**) variation in fruit shape and colour, (**c**) variable levels of pubescence. Accessions numbers are indicated.

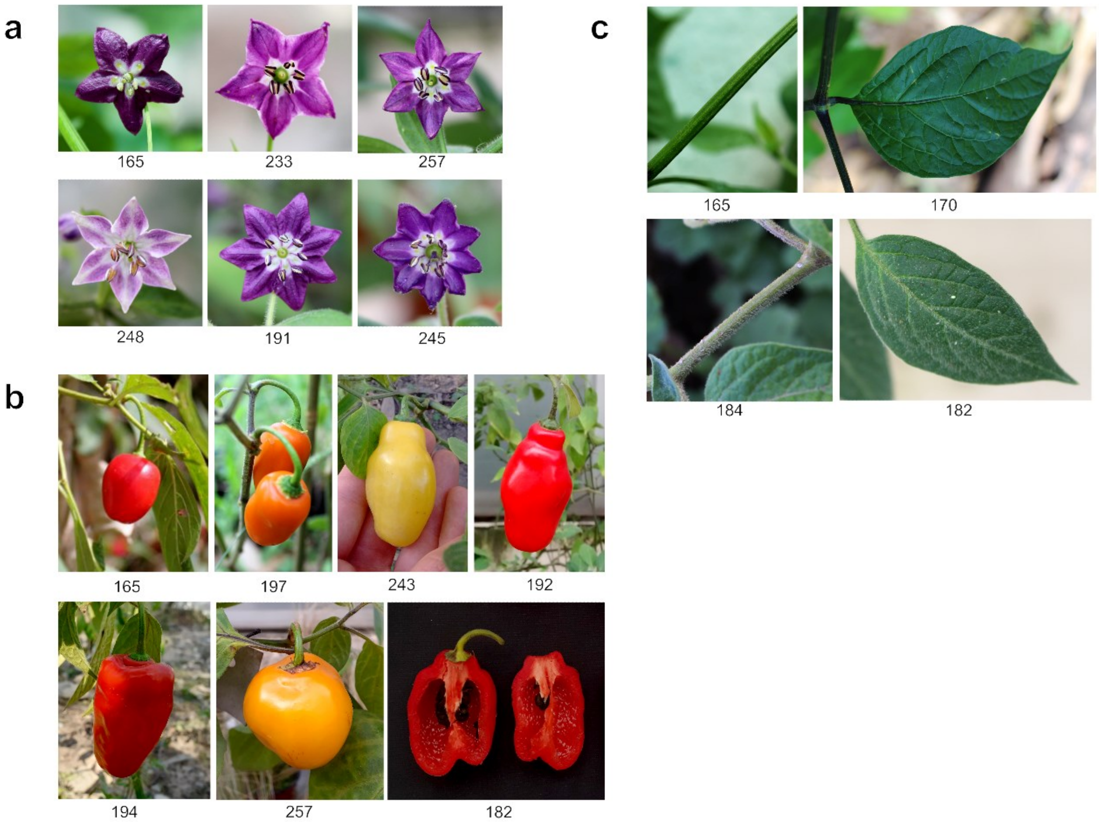

Figure S2. Results of the assessment of the sequence similarity clustering threshold parameter (ct) in ipyrad [61]. Values ranging from 0.85 to 0.99 were assessed. The number of clusters, the average read depth, the number of putatively paralogous loci (filtered by maxH), the allelic variation (heterozygosity), the number of retained loci, the retained sequence variation (number of SNPs), and the missingness percentage were recorded and plotted.

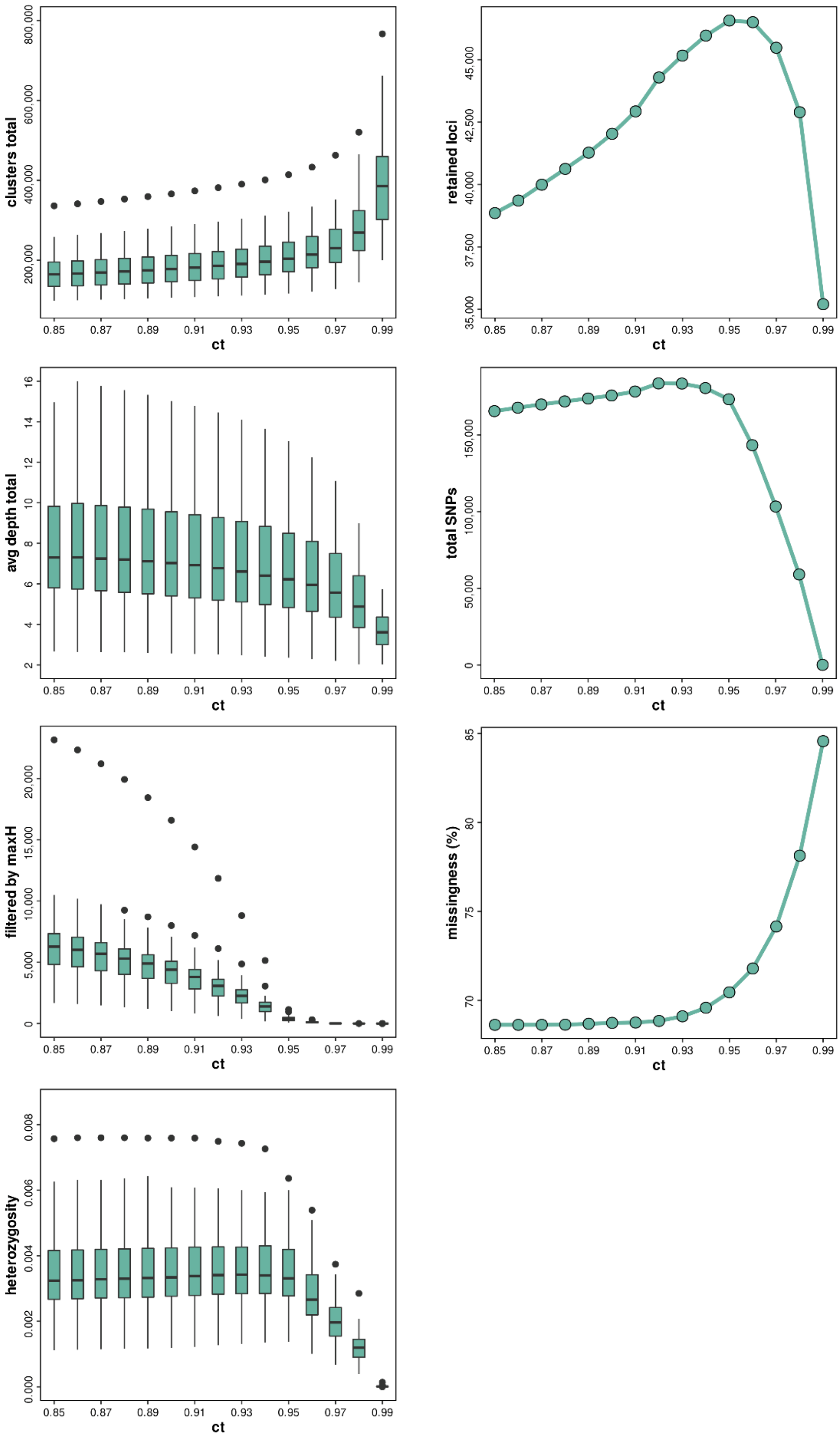

Table S1. Metadata for *C. pubescens* accessions analysed, and cluster assignment at *K*= 3.

ID	Locality and voucher number [if avalaible]	Origin	Fruit colour, shape	Raw reads	Filtered reads	Filtered loci at *m*= 4	Filtered loci at *m*= 34	DAPC cluster at *K*= 3	Admixture cluster at (*K*= 3)
202	Argentina: Jujuy, Humahuaca	local market	red, elongated	2634423	2377950	64845	42125	cluster 1	cluster 1
268	Argentina: Jujuy, Maimara	local market	yellow, elongated	1403257	1283816	46699	34903	cluster 1	cluster 1
269	Argentina: Jujuy, Maimara	local market	orange-red, blocky	1246268	1098685	26629	20894	cluster 1	cluster 1
64	Argentina: Salta, Salta	local market	orange-yellow, blocky	1375602	1298986	-	-	-	-
84	Argentina: Salta, Salta	local market	red, round	543647	534428	12727	11102	cluster 1	cluster 1
85	Argentina: Salta, Salta [Carrizo García (CG) 28]	local market	red, elongated	872440	858353	22245	19222	cluster 1	cluster 1
101	Argentina: Salta, Salta	local market	red, blocky	198626	192601	-	-	-	-
271	Argentina: Salta, Salta	local market	red	453973	564838	13595	11394	cluster 2	admixed
270	Argentina: Tucuman, San Miguel de Tucuman	seed donor	red, blocky	532181	834559	27185	21930	cluster 2	admixed
272	Argentina: Tucuman, San Miguel de Tucuman	seed donor	yellow, blocky	766317	916553	33543	26367	cluster 1	cluster 1
181	Bolivia: Chuquisaca, Muyupampa	local market	red, elongated	1925979	1830717	58670	39524	cluster 1	cluster 1
253	Bolivia: Chuquisaca, Muyupampa	local market	red, elongated	2091852	1956892	67906	42054	cluster 1	cluster 1
211	Bolivia: Chuquisaca, Campo Redondo [Barboza 4923]	family garden	unknown	2455388	2313256	64531	41722	cluster 1	admixed
190	Bolivia: Chuquisaca, Villa Serrano	local market	orange-yellow, elongated	2110489	1968839	61961	40677	cluster 2	cluster 2
191	Bolivia: Chuquisaca, Villa Serrano	local market	orange-yellow, elongated	1991526	1893578	62189	41161	cluster 2	admixed
192	Bolivia: Chuquisaca, Villa Serrano	local market	red, elongated	2126767	1982643	62085	40789	cluster 2	cluster 2
193	Bolivia: Chuquisaca, Villa Serrano	local market	red, elongated	2458157	1736772	46424	31032	cluster 2	admixed
179	Bolivia: Cochabamba	grocery store	red, blocky	1476390	1403666	51473	35928	cluster 1	cluster 1
245	Bolivia: Cochabamba, Cochabamba	grocery store	red, blocky	1759136	1633033	50128	35333	cluster 1	cluster 1
88	Bolivia: Cochabamba, Pojo	local market	red	257306	251084	-	-	-	-
198	Bolivia: Cochabamba, Pojo	local market	red, blocky	1627156	1556342	51309	35496	cluster 1	cluster 1
186	Bolivia: La Paz, La Paz ('yungueños')	local market	orange-red, round	2469779	2373189	61423	39414	cluster 2	cluster 2
187	Bolivia: La Paz, La Paz ('yungueños')	local market	orange-yellow, round	2298623	2120662	64485	41777	cluster 2	admixed

188	Bolivia: La Paz, La Paz (‘yungueños’)	local market	orange-yellow, round	3091143	2940865	67677	42883	cluster 2	admixed
189	Bolivia: La Paz, La Paz (‘yungueños’)	local market	red, elongated	1508887	1432400	42548	31125	cluster 2	cluster 2
243	Bolivia: La Paz, La Paz (‘yungueños’)	local market	light-yellow, elongated	2785501	1319629	51932	36731	cluster 2	cluster 2
259	Bolivia: La Paz, La Paz (‘yungueños’)	local market	red, elongated	956413	882998	30655	22595	cluster 2	admixed
196	Bolivia: La Paz, La Paz, (Achocalla)	local market	red, round	921611	870387	30475	23776	cluster 2	admixed
197	Bolivia: La Paz, La Paz, (Achocalla)	local market	orange, elongated	2362268	2041000	64059	40418	cluster 2	admixed
246	Bolivia: La Paz, La Paz, (Achocalla)	local market	orange-yellow, elongated	1223577	1159930	49539	36057	cluster 2	admixed
249	Bolivia: La Paz, La Paz, (Achocalla)	local market	red, round	2365979	2261278	64921	41915	cluster 2	admixed
9	Bolivia: La Paz, Apa Apa [Barboza et al. 3658]	growing freely in the field	orange-red, round	678175	669212	16037	13900	cluster 2	cluster 2
165	Bolivia: La Paz, Apa Apa [CG 75]	growing freely in the field	orange-red, round	1381022	1261441	41463	31495	cluster 2	cluster 2
172	Bolivia: La Paz, Coroico [Barboza 4890]	family garden	orange-yellow, round	932760	876737	35155	27487	cluster 2	cluster 2
173	Bolivia: La Paz, Coroico [Barboza 4890]	family garden	orange-yellow, round	719330	680765	11827	8948	cluster 2	cluster 2
174	Bolivia: La Paz, Coroico [Barboza 4890]	family garden	orange-yellow, round	1899800	1817337	53307	36989	cluster 2	cluster 2
170	Bolivia: La Paz, Huancane [Barboza 4889]	growing freely in the field	red, elongated	3813128	3662664	52667	34169	cluster 2	cluster 2
171	Bolivia: La Paz, Huancane [Barboza 4889]	growing freely in the field	red, round	948413	885680	23293	18215	cluster 2	cluster 2
286	Bolivia: La Paz, Huancane [Barboza 4889]	growing freely in the field	orange-yellow, round	404636	366227	3290	2645	cluster 2	cluster 2
89	Bolivia: Potosi, Tupiza	local market	red	575756	568595	-	-	-	-
199	Bolivia: Potosí, Tupiza	local market	red, elongated	1428175	1246406	44109	33081	cluster 1	admixed
184	Bolivia: Santa Cruz, Camiri	local market	red, blocky	2122192	1935005	57510	40230	cluster 1	cluster 1
185	Bolivia: Santa Cruz, Camiri	local market	red, elongated	1572988	1470334	43790	31169	cluster 1	admixed
194	Bolivia: Santa Cruz, Comarapa	local market	orange-yellow, blocky	1168069	1124773	34464	25980	cluster 1	cluster 1
195	Bolivia: Santa Cruz, Comarapa	local market	red, elongated	1630556	1503309	53022	36937	cluster 1	cluster 1
114	Bolivia: Santa Cruz, Moro Moro [CG et al. 35]	family garden	red, elongated	2146648	2044838	23850	17938	cluster 1	cluster 1
182	Bolivia: Santa Cruz, Moro Moro	family garden	red, elongated	753672	713598	19317	13334	cluster 1	cluster 1
83	Bolivia: Santa Cruz, Santa Cruz de la Sierra [CG 27]	local market	red	966666	952663	30604	24453	cluster 1	cluster 1
200	Bolivia: Santa Cruz, Santa Cruz de la Sierra	local market	red, elongated	1220486	1158638	42251	32077	cluster 1	cluster 1
102	Costa Rica	LP Co. ‘Costa Rican Red’	red, elongated	383611	379024	-	-	-	-
201	Costa Rica	LP Co. ‘Costa Rican Red’	red, elongated	1850043	1777304	54497	37136	cluster 3	cluster 3

91	Ecuador	LP Co. 'Ecuadorian Red'	red, elongated	892609	877893	29256	24227	cluster 2	admixed
256	Ecuador: Azuay	LP Co. 'Turbo pube'	orange-yellow, elongated	2576762	2387979	69684	42287	cluster 2	admixed
266	Ecuador: Loja	LP Co. 'Aji Largo'	red, elongated	1126367	998700	32416	25100	cluster 2	admixed
263	Guatemala	WUR 'CGN 22796'	red, blocky	834898	700821	14748	10714	cluster 2	cluster 3
257	Guatemala: Quiche, Santo Tomas Chichicastenango	LP Co. 'CAP 363'	orange-yellow, elongated	2585141	2431794	70572	42782	cluster 3	cluster 3
251	Mexico	LP Co. 'Manzano rojo'	red, blocky	1010453	933189	33496	25210	cluster 3	cluster 3
178	Mexico: Mexico City	local market	orange-yellow, blocky	1554713	1391440	47971	34231	cluster 3	cluster 3
203	Mexico: Mexico City	local market	orange-yellow, blocky	1272303	1210991	47019	34724	cluster 3	cluster 3
244	Mexico: Mexico City, Coyoacan	local market	orange-yellow, blocky	1133926	1085677	38233	29276	cluster 3	cluster 3
177	Mexico: Mexico City, Coyoacan	local market	orange-yellow, blocky	2586734	2299246	65751	41352	cluster 3	cluster 3
254	Peru	LP Co. 'CAP 217'	red, elongated	2657624	2441653	69825	42628	cluster 2	admixed
128	Peru: Cusco	local market	red, elongated	473582	466474	-	-	-	-
233	Peru: Cusco	local market	red, blocky	2098880	1847495	59975	39255	cluster 2	admixed
255	Peru: Cusco	local market	red, blocky	2092730	1941007	55417	37151	cluster 2	cluster 2
265	Peru: Huanuco	WUR 'CGN 22108'	red, elongated	1693733	1573667	56821	40467	cluster 2	admixed
262	Peru: Junin	WUR 'CGN 23768'	yellow, elongated	1012055	930939	35737	25589	cluster 2	admixed
90	Peru: Piura	local market	red	558605	551298	-	-	-	-
247	Peru: Piura	local market	red, blocky	1145483	1105495	38707	30610	cluster 2	admixed
175	Peru: Trujillo	local market	orange-yellow, blocky	2042003	1821689	61369	40018	cluster 2	admixed
176	Peru: Trujillo	local market	orange-yellow, blocky	1076414	999386	30560	23990	cluster 2	admixed
248	Spain: Canarias, LP (San Isidro)	LP Co. 'San Isidro'	orange-red, blocky	2169006	2025608	63998	41767	cluster 2	admixed
264	Unknown	seed donor	unknown	1143439	952540	21546	16535	cluster 3	cluster 3
267	Unknown	LP Co. 'Canario'	yellow, round	1402337	1127357	33528	25770	cluster 3	cluster 3

LP Co.: Semillas La Palma Company, Germany

WUR: genebank at the Centre for Genetic Resources, Wageningen University, The Netherlands

Table S2. Average Admixture cluster assignment for *C. pubescens* samples for *K*= 2 (**a**), 3 (**b**), 4 (**c**) and 5 (**d**) calculated from 1,462 unlinked biallelic SNPs markers.

(**a**) *K*= 2

Sample ID	Cluster 1	Cluster 2
114	1.0000	0.0000
165	0.8292	0.1708
170	0.6730	0.3270
171	0.6992	0.3008
172	0.5678	0.4322
173	0.6120	0.3880
174	0.6577	0.3423
175	0.0000	1.0000
176	0.0277	0.9723
177	0.0000	1.0000
178	0.0000	1.0000
179	0.8738	0.1262
181	1.0000	0.0000
182	1.0000	0.0000
184	1.0000	0.0000
185	1.0000	0.0000
186	0.3968	0.6032
187	0.3531	0.6469
188	0.4547	0.5453
189	0.5883	0.4117
190	0.7336	0.2664
191	0.8349	0.1651
192	0.7506	0.2494
193	0.7448	0.2552
194	1.0000	0.0000
195	1.0000	0.0000
196	0.6104	0.3896
197	0.4253	0.5747
198	1.0000	0.0000
199	0.7965	0.2035
200	1.0000	0.0000
201	0.0000	1.0000
202	1.0000	0.0000
203	0.0000	1.0000
211	0.9993	0.0007
233	0.0245	0.9755
243	0.2962	0.7038
244	0.0000	1.0000
245	0.8702	0.1298
246	0.5131	0.4869
247	0.0708	0.9292
248	0.0000	1.0000
249	0.4619	0.5381
251	0.0000	1.0000

253	1.0000	0.0000
254	0.0000	1.0000
255	0.1231	0.8769
256	0.0000	1.0000
257	0.0000	1.0000
259	0.5764	0.4236
262	0.0000	1.0000
263	0.0000	1.0000
264	0.0000	1.0000
265	0.0000	1.0000
266	0.0000	1.0000
267	0.0000	1.0000
268	0.8918	0.1082
269	0.8889	0.1111
270	0.0000	1.0000
271	0.0000	1.0000
272	1.0000	0.0000
286	0.5747	0.4253
83	1.0000	0.0000
84	1.0000	0.0000
85	1.0000	0.0000
9	0.6432	0.3568
91	0.0706	0.9294

(**b**) K= 3

Sample ID	Cluster 1	Cluster 2	Cluster 3
114	1.0000	0.0000	0.0000
165	0.3361	0.6639	0.0000
170	0.1973	0.7957	0.0070
171	0.1754	0.8246	0.0000
172	0.0000	0.9492	0.0507
173	0.0000	1.0000	0.0000
174	0.0608	0.9392	0.0000
175	0.0000	0.4492	0.5508
176	0.0000	0.6209	0.3791
177	0.0000	0.0000	1.0000
178	0.0000	0.0000	1.0000
179	0.9055	0.0092	0.0853
181	0.9197	0.0803	0.0000
182	1.0000	0.0000	0.0000
184	1.0000	0.0000	0.0000
185	0.6852	0.3148	0.0000
186	0.0000	0.8712	0.1288
187	0.1748	0.4998	0.3254
188	0.2588	0.5465	0.1947
189	0.0988	0.8836	0.0177
190	0.1755	0.8245	0.0000
191	0.3362	0.6546	0.0091
192	0.1485	0.8515	0.0000

193	0.3258	0.6742	0.0000
194	1.0000	0.0000	0.0000
195	0.9940	0.0000	0.0060
196	0.2319	0.7348	0.0333
197	0.2998	0.3901	0.3101
198	1.0000	0.0000	0.0000
199	0.6091	0.3909	0.0000
200	1.0000	0.0000	0.0000
201	0.0000	0.1121	0.8879
202	1.0000	0.0000	0.0000
203	0.0000	0.0000	1.0000
211	0.6641	0.3359	0.0000
233	0.0015	0.6269	0.3716
243	0.0000	0.8769	0.1231
244	0.0000	0.0000	1.0000
245	0.7559	0.2169	0.0273
246	0.3924	0.4523	0.1554
247	0.0070	0.5030	0.4900
248	0.0000	0.6028	0.3972
249	0.3405	0.4759	0.1836
251	0.0000	0.0000	1.0000
253	1.0000	0.0000	0.0000
254	0.0000	0.5419	0.4581
255	0.0000	0.7409	0.2591
256	0.0000	0.3710	0.6290
257	0.0000	0.0000	1.0000
259	0.3911	0.5017	0.1072
262	0.0000	0.3583	0.6417
263	0.0000	0.0452	0.9548
264	0.0000	0.0000	1.0000
265	0.0000	0.4635	0.5365
266	0.0000	0.5189	0.4811
267	0.0000	0.0000	1.0000
268	0.9704	0.0000	0.0296
269	0.9503	0.0000	0.0497
270	0.0000	0.4920	0.5080
271	0.0000	0.6599	0.3400
272	1.0000	0.0000	0.0000
286	0.0098	0.9295	0.0608
83	1.0000	0.0000	0.0000
84	1.0000	0.0000	0.0000
85	1.0000	0.0000	0.0000
9	0.2007	0.6917	0.1076
91	0.0825	0.3351	0.5824

(**c**) $K = 4$

Sample ID	Cluster 1	Cluster 2	Cluster 3	Cluster 4
114	0.9494	0.0427	0.0079	0.0000
165	0.2095	0.7537	0.0368	0.0000

170	0.0089	0.9574	0.0337	0.0000
171	0.0000	1.0000	0.0000	0.0000
172	0.0000	0.7582	0.2418	0.0000
173	0.0000	0.8642	0.1357	0.0000
174	0.0000	0.8216	0.1783	0.0000
175	0.0000	0.0000	0.8418	0.1582
176	0.0000	0.1466	0.7097	0.1437
177	0.0000	0.0000	0.0000	1.0000
178	0.0000	0.0000	0.0000	1.0000
179	0.8724	0.0000	0.1130	0.0147
181	0.8595	0.1382	0.0023	0.0000
182	1.0000	0.0000	0.0000	0.0000
184	1.0000	0.0000	0.0000	0.0000
185	0.5490	0.3528	0.0982	0.0000
186	0.0000	0.4501	0.4775	0.0725
187	0.1517	0.1895	0.4735	0.1853
188	0.2269	0.2143	0.4916	0.0671
189	0.0783	0.5545	0.3672	0.0000
190	0.0000	0.6000	0.4000	0.0000
191	0.0978	0.6019	0.2998	0.0005
192	0.0000	0.6000	0.4000	0.0000
193	0.1113	0.5558	0.3329	0.0000
194	0.8627	0.1373	0.0000	0.0000
195	0.9853	0.0000	0.0000	0.0146
196	0.2017	0.4100	0.3818	0.0065
197	0.2840	0.1275	0.4030	0.1855
198	0.9271	0.0729	0.0000	0.0000
199	0.5469	0.2987	0.1544	0.0000
200	1.0000	0.0000	0.0000	0.0000
201	0.0000	0.0147	0.2364	0.7488
202	1.0000	0.0000	0.0000	0.0000
203	0.0000	0.0000	0.0000	1.0000
211	0.5889	0.2963	0.1147	0.0000
233	0.0000	0.0379	0.9000	0.0621
243	0.0000	0.4285	0.5174	0.0541
244	0.0000	0.0000	0.0000	1.0000
245	0.7333	0.1617	0.0835	0.0214
246	0.3826	0.0940	0.4905	0.0328
247	0.0013	0.0269	0.7433	0.2285
248	0.0000	0.0981	0.6939	0.2079
249	0.3288	0.0562	0.5819	0.0331
251	0.0000	0.0000	0.0000	1.0000
253	1.0000	0.0000	0.0000	0.0000
254	0.0000	0.0972	0.6825	0.2203
255	0.0000	0.0503	0.9000	0.0497
256	0.0000	0.0000	0.6667	0.3333
257	0.0000	0.0000	0.0000	1.0000
259	0.3380	0.2560	0.3847	0.0212
262	0.0000	0.0556	0.5678	0.3766

263	0.0000	0.0000	0.2605	0.7395
264	0.0000	0.0000	0.0000	1.0000
265	0.0000	0.0000	0.8387	0.1613
266	0.0000	0.0779	0.6643	0.2578
267	0.0000	0.0000	0.0000	1.0000
268	0.9990	0.0000	0.0000	0.0010
269	0.9902	0.0000	0.0000	0.0097
270	0.0000	0.0298	0.6966	0.2736
271	0.0000	0.0440	0.9000	0.0561
272	0.9863	0.0137	0.0000	0.0000
286	0.0000	0.8009	0.1848	0.0143
83	1.0000	0.0000	0.0000	0.0000
84	0.9352	0.0520	0.0128	0.0000
85	0.9991	0.0000	0.0009	0.0000
9	0.0556	0.7624	0.0534	0.1286
91	0.0160	0.0000	0.6940	0.2900

(**d**) *K*= 5

**Sample ID**	**Cluster 1**	**Cluster 2**	**Cluster 3**	**Cluster 4**	**Cluster 5**
114	0.9314	0.0685	0.0000	0.0000	0.0000
165	0.1599	0.3660	0.4741	0.0000	0.0000
170	0.0112	0.3409	0.6479	0.0000	0.0000
171	0.0000	0.1810	0.8190	0.0000	0.0000
172	0.0000	0.0891	0.8558	0.0449	0.0102
173	0.0000	0.2671	0.6137	0.1178	0.0014
174	0.0000	0.0852	0.9147	0.0000	0.0000
175	0.0000	0.0000	0.0000	1.0000	0.0000
176	0.0000	0.0213	0.2039	0.7346	0.0402
177	0.0000	0.0000	0.0000	0.0000	1.0000
178	0.0000	0.0000	0.0000	0.0000	1.0000
179	0.8511	0.0000	0.0000	0.1488	0.0000
181	0.7922	0.0306	0.1772	0.0000	0.0000
182	1.0000	0.0000	0.0000	0.0000	0.0000
184	1.0000	0.0000	0.0000	0.0000	0.0000
185	0.3767	0.0583	0.5650	0.0000	0.0000
186	0.0000	0.0531	0.7166	0.1767	0.0536
187	0.1527	0.0867	0.1102	0.5376	0.1129
188	0.2302	0.0404	0.1618	0.5590	0.0086
189	0.0000	0.0498	0.8358	0.1143	0.0000
190	0.0000	1.0000	0.0000	0.0000	0.0000
191	0.0000	1.0000	0.0000	0.0000	0.0000
192	0.0000	1.0000	0.0000	0.0000	0.0000
193	0.0000	1.0000	0.0000	0.0000	0.0000
194	0.8061	0.0205	0.1734	0.0000	0.0000
195	0.9696	0.0000	0.0000	0.0000	0.0303
196	0.1712	0.0342	0.5248	0.2699	0.0000
197	0.2773	0.0641	0.0715	0.4566	0.1306
198	0.8386	0.0175	0.1439	0.0000	0.0000
199	0.5348	0.1913	0.1499	0.1239	0.0000

200	1.0000	0.0000	0.0000	0.0000	0.0000
201	0.0000	0.0000	0.0350	0.3020	0.6630
202	1.0000	0.0000	0.0000	0.0000	0.0000
203	0.0000	0.0000	0.0000	0.0000	1.0000
211	0.5756	0.3354	0.0889	0.0000	0.0000
233	0.0000	0.0000	0.0000	1.0000	0.0000
243	0.0000	0.0359	0.6716	0.2829	0.0096
244	0.0000	0.0000	0.0000	0.0000	1.0000
245	0.6928	0.0253	0.2207	0.0372	0.0240
246	0.3819	0.0533	0.0260	0.5388	0.0000
247	0.0000	0.0000	0.0000	0.9072	0.0928
248	0.0000	0.0101	0.1230	0.7210	0.1459
249	0.3382	0.0009	0.0000	0.6609	0.0000
251	0.0000	0.0000	0.0000	0.0000	1.0000
253	1.0000	0.0000	0.0000	0.0000	0.0000
254	0.0000	0.0000	0.0599	0.8466	0.0935
255	0.0000	0.0000	0.0000	1.0000	0.0000
256	0.0000	0.0000	0.0000	0.7812	0.2188
257	0.0000	0.0000	0.0000	0.0000	1.0000
259	0.3316	0.0968	0.1606	0.4110	0.0000
262	0.0000	0.0000	0.0238	0.7034	0.2728
263	0.0000	0.0000	0.0000	0.3186	0.6814
264	0.0000	0.0000	0.0000	0.0000	1.0000
265	0.0000	0.0000	0.0000	1.0000	0.0000
266	0.0000	0.0000	0.0659	0.7707	0.1634
267	0.0000	0.0000	0.0000	0.0000	1.0000
268	0.9992	0.0000	0.0000	0.0007	0.0000
269	0.9995	0.0000	0.0000	0.0000	0.0004
270	0.0000	0.0000	0.0000	0.8535	0.1465
271	0.0000	0.0000	0.0000	1.0000	0.0000
272	0.9170	0.0000	0.0830	0.0000	0.0000
286	0.0000	0.4398	0.4603	0.0999	0.0000
83	1.0000	0.0000	0.0000	0.0000	0.0000
84	0.8461	0.0924	0.0615	0.0000	0.0000
85	1.0000	0.0000	0.0000	0.0000	0.0000
9	0.0447	0.3750	0.4548	0.0000	0.1255
91	0.0000	0.0000	0.0000	0.8546	0.1454

Table S3. Common measures of genetic diversity for 67 *C. pubescens* samples from the inferred clusters at *K*= 2,4,5 calculated from 1,462 unlinked biallelic SNPs markers following (**a**) Admixture and (**b**) DAPC assignments, and also (**c**) Admixture n-balanced calculations. Samples origin/Clusters are named according to phylogenetic network grouping (Figure 2a) (see Table footers).

N = number of individuals; %P = percentage of polymorphic SNPs; A = total number of alleles; A_R = allelic richness, A_P = private allelic richness; H_O = observed heterozygosity; H_E = expected heterozygosity; F_{IS} = inbreeding coefficient; F_{IS} (95% CI) = lower and upper 95% confidence intervals of inbreeding coefficients.

(**a**) Admixture assignment

K-value	Cluster	N	%P	A	A_R	A_P	H_O	H_E	F_{IS}	F_{IS} (95% CI)
**2**	1	27	96.54	2819	1.7110	0.2840	0.2168	0.2106	0.0044	-0.0217, 0.0066
	2	25	87.57	2557	1.5842	0.1573	0.1265	0.1647	0.1856	0.1480, 0.2518
**4**	1	18	90.31	2619	1.1971	0.1172	0.2025	0.1905	-0.0178	-0.0460, -0.0111
	2	8	84.07	2438	1.2281	0.1329	0.2374	0.2053	-0.0478	-0.0728, -0.0221
	3	10	80.31	2329	1.1767	0.0800	0.1448	0.1661	0.1383	0.1006, 0.2037
	4	10	70.31	2039	1.1155	0.0642	0.1020	0.1084	0.1011	0.0816, 0.1698
**5**	1	17	89.18	2604	1.1944	0.0912	0.1996	0.1874	-0.0181	-0.0452, -0.0088
	2	4	73.15	2136	1.1941	0.0912	0.2331	0.1671	-0.2141	-0.2778, -0.2172
	3	5	84.73	2474	1.2603	0.1245	0.2873	0.2278	-0.1111	-0.1514, -0.0997
	4	14	83.63	2442	1.1782	0.0586	0.1373	0.1707	0.1793	0.1488, 0.2279
	5	8	66.51	1942	1.1019	0.0451	0.0955	0.0943	0.0511	0.0156, 0.1231

Samples origin/Clusters: *K*= 2- Cluster 1: C-S Bolivia-Argentina, Villa Serrano (Bolivia), C-W Bolivia; Cluster 2: Peru-Ecuador, Central America-Mexico. *K*= 4- Cluster 1: C-S Bolivia-Argentina; Cluster 2: C-W Bolivia (La Paz surroundings); Cluster 3: Peru-Ecuador; Cluster 4: Central America-Mexico. *K*= 5- Cluster 1: C-S Bolivia-Argentina, Cluster 2: Villa Serrano (Bolivia); Cluster 3: C-W Bolivia (La Paz surroundings); Cluster 4: Peru-Ecuador; Cluster 5: Central America-Mexico.

(**b**) DAPC assignment

K-value	Cluster	N	%P	A	A_R	A_P	H_O	H_E	F_{IS}	F_{IS} (95% CI)
**2**	1	40	99.18	2900	1.7662	0.2805	0.2203	0.2196	0.0197	0.0010, 0.0245
	2	27	90.46	2645	1.6071	0.1273	0.1389	0.1721	0.1486	0.1366, 0.1893
**4**	1	21	93.43	2732	1.2052	0.1160	0.2122	0.1993	-0.0151	-0.0616, -0.0162
	2	4	73.12	2138	1.1939	0.1129	0.2328	0.1669	-0.2141	-0.3000, -0.2119
	3	33	97.57	2853	1.2104	0.1009	0.1896	0.2065	0.0807	0.0266, 0.1592
	4	9	69.46	2031	1.1118	0.0662	0.1008	0.1044	0.0843	0.0729, 0.1204
**5**	1	21	93.43	2732	1.2052	0.0943	0.2122	0.1993	-0.0151	-0.0500, -0.0198
	2	4	73.12	2138	1.1939	0.0917	0.2328	0.1669	-0.2141	-0.2800, -0.2180
	3	17	95.69	2798	1.2325	0.0951	0.2388	0.2238	-0.0239	-0.0433, -0.0133
	4	16	86.11	2518	1.1794	0.0593	0.1417	0.1727	0.1625	0.1506, 0.2038
	5	9	69.46	2031	1.1118	0.0491	0.1008	0.1044	0.0843	0.0549, 0.1535

Samples origin/Clusters: *K*= 2- Cluster 1: C-S Bolivia–Argentina, Villa Serrano (Bolivia), C-W Bolivia; Cluster 2: Peru-Ecuador, Central America-Mexico. *K*= 4- Cluster 1: C-S Bolivia-Argentina; Cluster 2: Villa Serrano (Bolivia); Cluster 3: Peru-Ecuador, C-W Bolivia; Cluster 4: Central America-Mexico. *K*= 5- Cluster 1: C-S Bolivia–Argentina, Cluster 2: Villa Serrano (Bolivia); Cluster 3: C-W Bolivia; Cluster 4: Peru-Ecuador, C-W Bolivia (La Paz markets); Cluster 5: Central America-Mexico.

(**c**) Admixture n-balanced

K-value	Cluster	N	%P	A	A_R	A_P	H_O	H_E	F_{IS}	F_{IS} (95% CI)
**2**	1	27	96.54	2819	1.7110	0.2840	0.2168	0.2106	0.0044	-0.0217, 0.0066
	2	25	87.57	2557	1.5842	0.1573	0.1265	0.1647	0.1856	0.1480, 0.2518
**3**	1	10	85.07	2455	1.3987	0.1594	0.2075	0.1887	-0.0268	-0.0594, -0.0144
	2	10	92.76	2677	1.5105	0.2253	0.2630	0.2388	-0.0324	-0.0554, -0.0161
	3	10	70.76	2042	1.2349	0.0749	0.1065	0.1113	0.0880	0.0661, 0.1550
**4**	1	21	93.43	2732	1.2052	0.1160	0.2122	0.1993	-0.0151	-0.0616, -0.0162
	2	4	73.12	2138	1.1939	0.1129	0.2328	0.1669	-0.2141	-0.3000, -0.2119
	3	33	97.57	2853	1.2104	0.1009	0.1896	0.2065	0.0807	0.0266, 0.1592
	4	9	69.46	2031	1.1118	0.0662	0.1008	0.1044	0.0843	0.0729, 0.1204
**5**	1	8	81.40	2364	1.1931	0.0913	0.2046	0.1790	-0.0478	-0.0875, -0.0429
	2	4	73.24	2127	1.1935	0.0886	0.2315	0.1664	-0.2088	-0.1743, -0.2253
	3	8	83.71	2431	1.2268	0.1017	0.2364	0.2041	-0.0498	-0.0733, -0.0246
	4	8	78.37	2276	1.1743	0.0619	0.1247	0.1620	0.2277	0.1658, 0.2676
	5	8	66.87	1942	1.1049	0.0500	0.0979	0.0971	0.0538	0.0207, 0.1262

Samples origin/Clusters: *K*= 2- Cluster 1: C-S Bolivia-Argentina, Villa Serrano (Bolivia), C-W Bolivia; Cluster 2: Peru-Ecuador, Central America-Mexico. *K*= 3- Cluster 1: C-S Bolivia–Argentina; Cluster 2: Villa Serrano (Bolivia), C-W Bolivia, Peru-Ecuador; Cluster 3: Central America-Mexico; *K*= 4- Cluster 1: C-S Bolivia-Argentina; Cluster 2: C-W Bolivia (La Paz surroundings); Cluster 3: Peru-Ecuador; Cluster 4: Central America-Mexico. *K*= 5- Cluster 1: C-S Bolivia-Argentina, Cluster 2: Villa Serrano (Bolivia); Cluster 3: C-W Bolivia (La Paz surroundings); Cluster 4: Peru-Ecuador; Cluster 5: Central America-Mexico.

Table S4. Pairwise genetic differentiation (F_{ST}) among the inferred clusters of *C. pubescens* at *K*= 2,4,5 calculated from 1,462 unlinked biallelic SNPs markers following (**a**) Admixture and (**b**) DAPC assignments, and also (**c**) Admixture n-balanced calculations. Samples origin/Clusters are named according to phylogenetic network grouping (Figure 2a) (see Table footers).

(**a**) Admixture assignment

K= 2	Cluster 1	Cluster 2
**Cluster 1**	-	0.0776 - 0.0908
**Cluster 2**	0.0840	-

Cluster 1: C-S Bolivia-Argentina, Villa Serrano (Bolivia), C-W Bolivia; Cluster 2: Peru-Ecuador, Central

K= 4	Cluster 1	Cluster 2	Cluster 3	Cluster 4
**Cluster 1**	-	0.1364 – 0.1738	0.0739 – 0.0907	0.2202 – 0.2614
**Cluster 2**	0.1553	-	0.1043 – 0.1417	0.3096 – 0.3605
**Cluster 3**	0.0817	0.1233	-	0.1158 – 0.1479
**Cluster 4**	0.2404	0.3368	0.1322	-

Cluster 1: C-S Bolivia-Argentina; Cluster 2: C-W Bolivia (La Paz surroundings); Cluster 3: Peru-Ecuador; Cluster 4: Central America-Mexico

K= 5	Cluster 1	Cluster 2	Cluster 3	Cluster 4	Cluster 5
**Cluster 1**	-	0.1396 – 0.1711	0.0644 – 0.0780	0.1084 – 0.1282	0.2231 – 0.2573
**Cluster 2**	0.1553	-	0.1044 – 0.1334	0.1479 – 0.1840	0.3141 – 0.3583
**Cluster 3**	0.0711	0.1189	-	0.0381 – 0.0507	0.1554 – 0.1836
**Cluster 4**	0.1183	0.1651	0.0441	-	0.1300 – 0.1612
**Cluster 5**	0.2404	0.3368	0.1692	0.1460	-

Cluster 1: C-S Bolivia-Argentina, Cluster 2: Villa Serrano (Bolivia); Cluster 3: C-W Bolivia (La Paz surroundings); Cluster 4: Peru-Ecuador; Cluster 5: Central America-Mexico.

(**b**) DAPC assignment

K= 2	1	2
**1**	-	0.0776 - 0.0908
**2**	0.0840	-

Cluster 1: C-S Bolivia–Argentina, Villa Serrano (Bolivia), C-W Bolivia; Cluster 2: Peru-Ecuador, Central America-Mexico.

K= 4	Cluster 1	Cluster 2	Cluster 3	Cluster 4
**Cluster 1**	-	0.1364 – 0.1738	0.0739 – 0.0907	0.2202 – 0.2614
**Cluster 2**	0.1553	-	0.1043 – 0.1417	0.3096 – 0.3605
**Cluster 3**	0.0817	0.1233	-	0.1158 – 0.1479
**Cluster 4**	0.2404	0.3368	0.1322	-

Cluster 1: C-S Bolivia-Argentina; Cluster 2: Villa Serrano (Bolivia); Cluster 3: Peru-Ecuador, C-W Bolivia; Cluster 4: Central America-Mexico.

K= 5	Cluster 1	Cluster 2	Cluster 3	Cluster 4	Cluster 5
**Cluster 1**	-	0.1396 – 0.1711	0.0644 – 0.0780	0.1084 – 0.1282	0.2231 – 0.2573
**Cluster 2**	0.1553	-	0.1044 – 0.1334	0.1479 – 0.1840	0.3141 – 0.3583
**Cluster 3**	0.0711	0.1189	-	0.0381 – 0.0507	0.1554 – 0.1836
**Cluster 4**	0.1183	0.1651	0.0441	-	0.1300 – 0.1612
**Cluster 5**	0.2404	0.3368	0.1692	0.1460	-

Cluster 1: C-S Bolivia–Argentina, Cluster 2: Villa Serrano (Bolivia); Cluster 3: C-W Bolivia; Cluster 4: Peru-Ecuador, C-W Bolivia (La Paz markets); Cluster 5: Central America-Mexico.

(**c**) Admixture n-balanced

K= 2	Cluster 1	Cluster 2
**Cluster 1**	-	0.1105 – 0.1273
**Cluster 2**	0.1189	-

Cluster 1: C-S Bolivia-Argentina, Villa Serrano (Bolivia), C-W Bolivia; Cluster 2: Peru-Ecuador, Central America-Mexico.

K= 2	Cluster 1	Cluster 2	Cluster 3
**Cluster 1**	-	0.0855 – 0.1001	0.2330 – 0.2672
**Cluster 2**	0.0933	-	0.1578 – 0.1860
**Cluster 3**	0.2501	0.1718	-

Cluster 1: C-S Bolivia–Argentina; Cluster 2: Villa Serrano (Bolivia), C-W Bolivia, Peru-Ecuador; Cluster 3: Central America-Mexico.

K= 4	Cluster 1	Cluster 2	Cluster 3	Cluster 4
**Cluster 1**	-	0.1074 - 0.1464	0.1240 – 0.1464	0.2324 – 0.2674
**Cluster 2**	0.1181	-	0.1010 – 0.1274	0.2390 – 0.2742
**Cluster 3**	0.1351	0.1708	-	0.1527 – 0.1878
**Cluster 4**	0.2501	0.2569	0.1708	-

Cluster 1: C-S Bolivia-Argentina; Cluster 2: C-W Bolivia (La Paz surroundings); Cluster 3: Peru-Ecuador; Cluster 4: Central America-Mexico.

K= 5	Cluster 1	Cluster 2	Cluster 3	Cluster 4	Cluster 5
**Cluster 1**	-	0.1597 - 0.1923	0.1267 - 0.1497	0.1220 - 0.1434	0.2519 - 0.2902
**Cluster 2**	0.1761	-	0.1501 - 0.1848	0.2421 - 0.2776	0.3350 - 0.3791
**Cluster 3**	0.1377	0.1662	-	0.0709 - 0.0938	0.1501 - 0.1871
**Cluster 4**	0.1321	0.2595	0.0826	-	0.1452 - 0.1769
**Cluster 5**	0.2711	0.3569	0.1681	0.1616	-

Cluster 1: C-S Bolivia-Argentina, Cluster 2: Villa Serrano (Bolivia); Cluster 3: C-W Bolivia (La Paz surroundings); Cluster 4: Peru-Ecuador; Cluster 5: Central America-Mexico.
